# Study of the Relationship Between the Number of Metabolic Syndrome Components and Subclinical Cardiac Dysfunction: A Retrospective Analysis

**DOI:** 10.3390/jcm15082920

**Published:** 2026-04-11

**Authors:** Monika Starzak, Grzegorz K. Jakubiak, Natalia Pawlas, Grzegorz Cieślar, Agata Stanek

**Affiliations:** 1 Department of Internal Medicine, Angiology and Physical Medicine, Faculty of Medical Sciences in Zabrze, Medical University of Silesia in Katowice, Batorego 15 St., 41-902 Bytom, Poland; cieslar1@tlen.pl; 2 Department of Pharmacology, Faculty of Medical Sciences in Zabrze, Medical University of Silesia in Katowice, Jordana 38 St., 41-808 Zabrze, Poland; grzegorz.k.jakubiak@gmail.com (G.K.J.); n-pawlas@wp.pl (N.P.); 3 Department of Internal Medicine, Metabolic Diseases and Angiology, Faculty of Health Sciences in Katowice, Medical University of Silesia in Katowice, Ziolowa 45/47 St., 40-635 Katowice, Poland; astanek@tlen.pl

**Keywords:** metabolic syndrome, echocardiography, left ventricular mass, left atrial volume, subclinical cardiac dysfunction

## Abstract

**Background:** Metabolic syndrome (MetS) comprises coexisting risk factors enhancing the likelihood of developing cardiovascular disease (CVD). The aim of this study was to investigate the correlation between the number of MetS components and subclinical cardiac dysfunction, assessed via transthoracic echocardiography (TTE), in individuals without overt CVD. **Methods:** A retrospective analysis was performed using data from 100 patients (63% female; mean age 58.8 ± 16.81 years) hospitalized in the Department of Internal Medicine, Angiology and Physical Medicine at the Medical University of Silesia in Katowice, Poland, between June 2022 and February 2024. The inclusion criteria were the absence of diagnosed atherosclerotic CVD and no evidence of acute illness or exacerbation of chronic diseases. Each participant was evaluated for MetS components and underwent TTE. **Results:** Univariate analysis revealed significant correlations between the number of MetS components and selected TTE parameters, including left ventricular mass (LVM) (R = 0.406; *p* < 0.001), left ventricular mass index (LVMI) (R = 0.248; *p* = 0.013), left ventricular ejection fraction (LV EF) (R = −0.261; *p* = 0.009), left atrial volume (LAV) (R = 0.312; *p* < 0.001), and left atrial volume index (LAVI) (R = 0.273; *p* = 0.007). These correlations did not remain significant after adjusting for age, sex, and body mass index (BMI). Among patients not meeting the full diagnostic criteria for MetS, LAV and LAVI values remained significantly correlated with the number of MetS components, independent of confounding variables. **Conclusions:** The selected echocardiographic parameters were significantly correlated with the number of MetS components; however, most associations were explained by age, sex, and BMI.

## 1. Introduction

Metabolic syndrome (MetS) remains a significant contributor to the development of cardiovascular diseases (CVDs), including the onset and progression of heart failure (HF) [1]. Hemodynamic alterations linked to MetS increase the risk of developing HF with preserved left ventricular ejection fraction (HFpEF), which is primarily characterized by diastolic dysfunction [2,3]. CVDs continue to be a significant cause of morbidity and mortality. Early detection of these conditions remains essential [4,5]. Although MetS has various definitions, it generally refers to the coexistence of several modifiable risk factors that increase the risk of developing CVD [6,7]. The presence of specific, well-defined disorders substantially increases the risk of atherosclerotic CVDs, such as coronary artery disease [8].

The main components of MetS are abdominal obesity, elevated blood pressure, increased serum triglyceride (TG) concentrations, decreased serum high-density lipoprotein cholesterol (HDL-C) concentrations, and hyperglycemia [9]. While the diagnosis of MetS and its components is known to contribute to the development of left ventricular hypertrophy and an increased risk of HFpEF [10,11,12], the relationship between the number of MetS components and cardiac morphology and function, as assessed via echocardiography in individuals without overt CVD, remains unclear.

This study intended to examine the correlation between the MetS components count and the presence of subclinical cardiac dysfunction assessed via transthoracic echocardiography (TTE) in individuals without overt CVD.

## 2. Materials and Methods

### 2.1. Study Population

This study conducted a retrospective analysis of medical records from patients hospitalized in the Department of Internal Medicine, Angiology and Physical Medicine at the Medical University of Silesia in Katowice, Poland, between June 2022 and February 2024. The investigated population consisted of patients admitted for planned internal medicine diagnostics who were assessed for features of MetS and underwent TTE during hospitalization.

The exclusion criteria were as follows: (1) acute illness within one month prior to hospital admission; (2) a medical history of overt atherosclerotic CVD, including angiographically confirmed coronary artery disease with hemodynamically significant stenoses, previous myocardial infarction, prior angioplasty of coronary arteries or other vascular beds, previous surgical revascularization of the myocardium or other vascular beds, or previous stroke; (3) prior implantation of a cardiac implantable device; (4) clinical symptoms of HF; (5) infection; (6) diagnosed autoimmune disease undergoing immunosuppressive treatment; (7) diagnosed malignant tumor; and (8) anemia.

### 2.2. Clinical and Laboratory Measurements

Waist circumference (WC) and hip circumference (HC) were assessed in a standing position. Body mass index (BMI) was calculated as weight in kilograms divided by height in meters squared. Overweight was established as BMI ≥ 25 kg/m^2^, and obesity was established as BMI ≥ 30 kg/m^2^. Body composition analysis was conducted using the bioelectrical impedance technique with a TANITA MC-780 device (Tanita Europe B.V., Amsterdam, Netherlands).

Blood pressure level and heart rate were quantified by an automatic oscillometric instrument while seated after a 10 min rest time. Arterial hypertension was established as systolic blood pressure of 140 mmHg or higher, diastolic blood pressure of 90 mmHg or higher, or a prior clinical diagnosis of hypertension with ongoing treatment with antihypertensive agents.

Diabetes mellitus was defined in accordance with the diagnostic criteria published by the American Diabetes Association or by a documented history of diabetes diagnosis and the use of antidiabetic medications [13].

Blood samples for laboratory analysis were collected between 6:00 and 8:00 a.m. after a minimum 14 h fasting period. All laboratory analyses were carried out at the Laboratory of the Specialist Hospital No. 2 in Bytom.

### 2.3. Definition of Metabolic Syndrome

MetS is defined by the 2009 consensus of the International Diabetes Federation in collaboration with the National Heart, Lung, and Blood Institute; American Heart Association; World Heart Federation; International Atherosclerosis Society; and International Association for the Study of Obesity. According to this consensus, MetS is recognized if meeting a minimum of three out of five criteria: (1) waist circumference (WC) ≥ 94 cm in men or ≥80 cm in women; (2) TG level ≥ 150 mg/dL or drug treatment for elevated TG level; (3) HDL-C < 40 mg/dL in men or <50 mg/dL in women or drug treatment for reduced HDL-C; (4) systolic blood pressure ≥ 130 mmHg and/or diastolic blood pressure ≥ 85 mmHg, or treatment with antihypertensive agents; and (5) preprandial serum glucose level ≥ 100 mg/dL or use of antidiabetic agents [14].

### 2.4. Transthoracic Echocardiography

TTE was conducted by an accredited cardiologist using a Vivid E9 device equipped with a 4 MHz sector-type M5S-D probe (GE HealthCare Ultrasound, Chicago, IL, USA), in accordance with current guidelines [15]. All examinations were conducted by the same physician.

Linear internal measurements of left ventricular end-diastolic diameter (LV EDD), left ventricular end-systolic diameter (LV ESD), interventricular septum thickness (IVS), and posterior wall thickness (PWT) were obtained from the parasternal long-axis view. Left ventricular mass was computed using the linear method and the given cube formula:

LV mass = 0.8 × [1.04 × (LV EDD + IVS + PWT)^3^ − (LV EDD)^3^] + 0.6 g (1)

Left ventricular mass index (LVMI) was derived as the ratio of left ventricular mass (LVM) to body surface area, with body surface area estimated using the de Buois formula.

Left ventricular (LV) systolic function was evaluated using left ventricular ejection fraction (LV EF) and left ventricular fractional shortening (LV FS). LV EF was determined using Simpson’s two-dimensional biplane method.

Left atrial size was measured from the parasternal long-axis view, as well as the apical four- and two-chamber views. Left atrial volume (LAV) was adjusted for body surface area to derive the left atrial volume index (LAVI).

Doppler examination was conducted from the apical four-chamber view. Peak velocities of the early (E) and late (A) phases of mitral inflow were acquired. Tissue Doppler imaging was used to assess peak early diastolic velocities (E′ septal and E′ lateral) and peak systolic velocities (S′ septal and S′ lateral) of the mitral valve annulus motion.

### 2.5. Statistical Analyses

For quantitative variables, the normality of each variable was assessed using the Kolmogorov–Smirnov and Shapiro–Wilk tests, as well as visual inspection of histograms. Variables with a distribution that showed no significant difference from the normal distribution are presented using means and standard deviations (SDs). Variables with distributions that differed significantly from normality are presented using medians and interquartile ranges. Qualitative variables are presented as the number of each variable variant and the percentage. The Spearman rank correlation test was employed to assess the association between quantitative variables. A *p*-value less than 0.05 was considered significant at the statistical level.

Following univariate analysis of correlations, a multivariate model was constructed to elucidate the relationship between the number of MetS components and selected echocardiographic parameters while controlling for confounding variables. The model included adjustments for age, sex, and BMI. Non-normally distributed variables were log-transformed prior to multivariate analysis to better approximate normality and meet model assumptions.

Missing data were minimal and handled using a complete-case (listwise deletion) approach. Given the low proportion of missing values, this method was considered unlikely to introduce significant bias or affect the robustness of the results.

Due to the exploratory nature of the study and the limited sample size, no formal correction for multiple testing was implemented. Including multiple corrections in small datasets may increase the risk of type II error and obscure potentially meaningful associations. Therefore, the results were interpreted with caution, taking into account effect sizes, consistency, and clinical relevance.

Statistical analyses were carried out using TIBCO Software Inc., Palo Alto, CA, USA (2017), Statistica (data analysis software system), version 13.

### 2.6. Ethical Aspects

The present study is based on a retrospective data analysis and therefore did not require approval from a bioethics committee. This was confirmed by the Bioethics Committee of the Medical University of Silesia in Katowice (6 February 2024, BNW/NWN/0052/KB/19/24).

## 3. Results

### 3.1. Characteristics of the Study Population

The final analysis comprised 100 patients. Among them, 29 (29.0%) had obesity, and 35 (35.0%) had overweight. The basic characteristics of the investigated population are shown in Table 1.

A total of 45 patients (45.0%) met the criteria for MetS. The number of individual subgroups depending on the number of MetS components is shown in Table 2.

Patients with MetS were significantly older (62.94 ± 11.63 vs. 55.41 ± 19.54 years; *p* = 0.025) and characterized by a higher BMI (29.8 ± 4.43 vs. 24.73 ± 4.28 kg/m^2^; *p* < 0.001), WC (102.33 ± 11.66 vs. 86.52 ± 13.76 cm; *p* < 0.001), and waist-to-hip ratio (WHR) (0.95 ± 0.09 vs. 0.88 ± 0.09; *p* < 0.001). Moreover, patients with MetS were characterized by a significantly higher body fat mass (27.36 ± 8.07 vs. 19.48 ± 7.4 kg; *p* < 0.001) and fat percentage (32.17 ± 6.39 vs. 27.97 ± 7.95%; *p* = 0.005).

### 3.2. Echocardiographic Parameters

In most cases, TTE did not reveal any significant abnormalities. LV EF ≤ 50% was found in 10% of the study population, with LV EF ≤ 45% found in only 3%. LAVI > 34 mL/m^2^ was found in 15.15% of the study population. TRV_max_ > 2.8 m/s was found in only three patients. E/E′ average > 14.0 was found in 3% of the study population. Full descriptive statistics relating to echocardiographic parameter values are presented in Table 3.

### 3.3. Association of Echocardiographic Parameters with the Number of Metabolic Syndrome Components

In the study population, statistically significant positive correlations were found between the number of MetS components and left ventricular width (LV EDD and LV ESD) and all parameters related to left ventricular myocardial mass (IVS, PWT, LVM, and LVMI). Furthermore, the number of MetS components was found to be negatively correlated with left ventricular systolic function when assessed via LV EF but not when assessed via LV FS. Moreover, the number of MetS components was significantly positively correlated with parameters relating to the measurements of the left atrium (LA, LAV, and LAVI) and the right ventricle (RV D1). A significant negative association was found between the MetS components count and the E/A ratio. There was no statistical significant association of MetS component count and the average E/E′ or TRV_max_.

All results pertaining to the correlations between the number of MetS components and the values of echocardiographic parameters are presented in Table 4.

### 3.4. Multivariate Analysis

Based on the multivariate analysis conducted, the constructed model was significant for the following echocardiographic parameters: LV EDD, LV ESD, LVM, LVMI, LA, LAV, LAVI, log(RV D1), TRV_max_, log(E), log(A), log(E′ septal), log(E′ lateral), log(E/E′ septal), log(E/E′ lateral), and log(E/E′ average). The full results are presented in Table 5.

After performing the multivariate analysis adjusted for age, sex, and BMI, no significant relationship was found between MetS component count and the values of assessed echocardiographic parameters. The full results are shown in Table 6.

Additional analyses were conducted to examine the correlation of LAV and LAVI and the number of MetS components, separately for patients with MetS and those without MetS. In patients not meeting the full diagnostic criteria for MetS, the model was significant for both LAV and LAVI. In patients diagnosed with MetS, the model was significant only for LAV. The full results are shown in Table 7.

Based on the results of the multivariate analysis standardized for age, sex, and BMI, a relevant relationship was confirmed between LAV and LAVI and the number of MetS components, regardless of confounding variables, in patients not meeting the full diagnostic criteria for MetS. The full results are presented in Table 8.

## 4. Discussion

In recent decades, the understanding of the pathophysiology underlying CVD progression has advanced significantly. The early detection of CVD remains critical due to persistently high morbidity and mortality rates [16]. Recognition of metabolic disorders in cardiac risk stratification and disease development, as well as their reciprocal influence, is increasingly emphasized; consequently, the role of MetS is considered fundamental [17,18].

Our study found the number of MetS components to show significant correlation with measurements related to left ventricular mass (LVM and LVMI), left ventricular size (LV EDD and LV ESD), left ventricular systolic function (LV EF, but not LV FS), left atrial size (LA, LAV, and LAVI), and left ventricular diastolic dysfunction (E/A but not E/E′ average or TRV_max_), as assessed via TTE. However, after performing multivariate analysis adjusted for age, sex, and BMI, no significant relationship was confirmed between the MetS components count and the values of the assessed echocardiographic parameters. The attenuation of associations after adjustment for age, sex, and BMI reflects their confounding role, emphasizing the importance of obesity as the core of MetS. Furthermore, the correlation between LAV and LAVI and the number of MetS components retained significance after being standardized for age, sex, and body mass index (BMI), but only in patients who did not meet the full diagnostic criteria for MetS.

A key strength of our study is the examination of the relationship between the number of MetS components and echocardiographic parameters in individuals without overt CVD. While a majority of previous studies focused on the impact of individual disorders, our approach considers the cumulative effect of multiple components. Additionally, analyzing a cohort without overt CVD enables a more precise assessment of the association between the number of MetS components and subclinical alterations in cardiac morphology and function. The retrospective nature of this study may have introduced potential biases, including selection bias, and resulted in incomplete data collection; however, the exclusion criteria were carefully followed, thus allowing the focus to be placed on patients without overt CVD.

According to the latest studies on MetS, obesity is considered the primary metabolic disorder. Adipocytes are no longer considered to be an energy storage depot but rather active metabolic tissue, which, due to inflammatory mediators and hormones, leads to cardiac and vascular impairment [19,20,21]. Metabolic disorders promote hemodynamic changes, the endocrine and paracrine effects of adipose tissue, neurohormonal activation, lipotoxicity, and increased arterial stiffness, all of which contribute to left ventricular remodeling. Myocardial remodeling primarily increases the risk of HFpEF, as well as diastolic and subsequent systolic dysfunction, ultimately resulting in a progressive decline in myocardial function [22,23,24]. Early myocardial impairment may be assessed through myocardial strain and strain rate parameters measured via speckle-tracking echocardiography. Several studies have shown that left ventricular global longitudinal strain (LV-GLS) is impaired in patients with metabolic dysfunction independent of demographics, anthropometrics, and the CVD burden. Moderate-to-severe non-alcoholic fatty liver disease with concomitant type 2 diabetes mellitus leads to progressive deterioration in left ventricular strain parameters while preserving LVEF [25,26].

Obesity and overweight lead to higher cardiac output, which consequently results in LV dilatation and subsequently hypertrophy due to an increase in LV wall loading [27,28]. Pandey et al. analyzed three cohort studies including 51,451 participants and identified lower leisure time physical activity and higher BMI to be independent risk factors for HF [29]. Although epidemiological studies have shown a strong relationship between the development of HF and obesity, HF remains underdiagnosed and therefore undertreated in patients with excess body fat [30,31]. HF constitutes a major public health problem, with a worldwide prevalence of over 64 million. Early detection and target-directed therapy represent crucial clinical treatment strategies for this disorder. Over the years, the Framingham Heart Study and others have shown that MetS with obesity as the core feature is associated with at least a two-fold higher risk of the development of HF, independent of traditional cardiovascular risk factors, indicating the importance of lipotoxicity in HF progression [32]. The attenuation of associations after adjustment for BMI suggests that adiposity may represent a key integrative factor linking metabolic disturbances with cardiac remodeling. Rather than acting independently, individual components of MetS likely reflect overlapping biological pathways, such as inflammation, insulin resistance, and lipotoxicity, that converge on myocardial structure and function.

An interesting finding of our study is the significant relationship between LAV and LAVI and the number of MetS components, which is independent of age, sex, and BMI, but only in individuals not meeting the full diagnostic criteria for MetS. Similar findings related to myocardial mass were found in a study conducted by a research group from the United States (Apridonidze et al.). Left ventricular myocardial mass, expressed as the LV mass/height^2.7^ ratio, increased with the number of MetS components; however, the largest differences were observed in individuals with fewer than three MetS components [33]. In the aforementioned study, LAV and LAVI were not assessed, but it is worth noting that an increase in LVM promotes the development of left ventricular diastolic dysfunction, one of the structural determinants of which is an increase in LAVI.

### Study Limitations

The present study has several limitations. It does not allow for conclusions to be drawn regarding causal relationships but rather identifies correlations among specific parameters assessed at a single time point. Moreover, the study involved a relatively small patient cohort, which precluded additional subgroup analyses. Future research should replicate this study with a larger sample size. Furthermore, due to the retrospective nature of the study, some data were missing, although the extent was relatively minor. Another limitation of the study is the absence of advanced echocardiographic techniques, such as global longitudinal strain (GLS), which is an early biomarker of myocardial dysfunction. Future prospective studies incorporating speckle-tracking echocardiography are warranted to improve the detection of subtle cardiac abnormalities. Future research should focus on conducting a more precise assessment of metabolism, including the use of additional parameters related to insulin resistance and a more detailed evaluation of lipid metabolism. It is also worth repeating similar studies while taking into account different MetS definitions.

## 5. Conclusions

This study found that a majority of the assessed echocardiographic parameters related to cardiac morphology and function were significantly correlated with the number of MetS components in univariate analysis. Our findings suggest that the observed associations between the number of MetS components and cardiac parameters are largely influenced by established confounders such as age, sex, and BMI. Therefore, these results should be interpreted cautiously and considered hypothesis-generating. Further studies are needed to clarify whether MetS components exert independent effects on cardiac adaptation.

## Figures and Tables

**Table 1:** Basic characteristics of the investigated population.

Variable		N (%)/Mean ± SD/Median (Q1; Q3)
Age [years]		58.8 ± 16.81
Gender		Females: 63 (63.0%) Males: 37 (37.0%)
Hypertension		63 (63.0%)
Impaired carbohydrate metabolism	Diabetes	22 (22.0%)
	Prediabetes	21 (21.0%)
Smoking	Current smoker	43 (43.4%)
	Former smoker	27 (27.3%)
	Never	29 (29.3%)
Metabolic syndrome		45 (45.0%)
Chronic kidney disease		2 (2.0%)
Atrial fibrillation	Paroxysmal	1 (1.0%)
	Persistent	4 (4.0%)
Body mass index (BMI) [kg/m^2^]		27.01 ± 5.02
Waist-to-hip ratio (WHR)		0.91 ± 0.095
Waist-to-height ratio (WHtR)		0.56 ± 0.092
Fat mass [kg]		23.03 ± 8.62
Fat percentage [%]		29.86 ± 7.55
Total cholesterol (TC) [mg/dL]		189.67 ± 38.22
Low-density lipoprotein cholesterol (LDL-C) [mg/dL]		120.34 ± 35.18
High-density lipoprotein cholesterol (HDL-C) [mg/dL]		55.39 ± 14.64
Triglycerides (TGs) [mg/dL]		118.5 (78.0; 161.5)
Non-HDL-C [mg/dL]		131.85 (104.55; 158.5)
Uric acid [mg/dL]		5.0 (4.05; 6.3)
Glycated hemoglobin [%]		5.61 (5.37; 5.91)

**Table 2:** The number of individual subgroups depending on the number of metabolic syndrome components.

Number of Metabolic Syndrome Components	N (%)
0	12 (12.0%)
1	15 (15.0%)
2	28 (28.0%)
3	22 (22.0%)
4	13 (13.0%)
5	10 (10.0%)

**Table 3:** The values of echocardiographic parameters in the investigated population.

Parameter	Mean ± SD/Median (Q1; Q3)	Minimal Value	Maximal Value
Left ventricle parameters			
IVS [mm]	10.0 (10.0; 10.0)	6.0	13.0
PWT [mm]	10.0 (10.0; 10.0)	6.0	12.0
LV EDD [mm]	46.67 ± 5.42	34.0	61.0
LV ESD [mm]	35.0 ± 5.29	25.0	48.0
LVM [g]	160.34 ± 39.49	63.74	279.03
LVMI [g/m^2^]	87.33 ± 18.69	42.58	140.91
RWT	0.42 ± 0.064	0.262	0.556
LV SV [mL]	74.21 (62.64; 87.88)	38.15	183.48
LV EF [%]	56.0 (54.0; 59.0)	43.0	62.0
LV FS [%]	25.07 ± 6.37	11.63	41.82
S′ septal [cm/s]	9.0 (8.0; 10.0)	5.0	16.0
S′ lateral [cm/s]	9.0 (8.0; 11.0)	6.0	17.0
Left atrium parameters			
LA [mm]	32.98 ± 4.74	22.0	48.0
LAV [mL]	45.0 (36.3; 55.85)	22.04	104.72
LAVI [mL/m^2^]	24.54 (19.61; 29.37)	12.52	51.51
Right ventricle parameters			
RV D1 [mm]	31.0 (28.0; 34.5)	22.0	41.0
TAPSE [mm]	24.0 (23.0; 24.0)	10.0	27.0
Diastolic function parameters			
TRV_max_ [m/s]	2.1 ± 0.38	1.3	3.0
AcT [ms]	100.5 (92.5; 109.5)	60.0	152.0
E [m/s]	0.7 (0.6; 0.8)	0.4	1.6
A [m/s]	0.7 (0.6; 0.8)	0.3	1.3
E/A	1.0 (0.71; 1.33)	0.53	2.33
E′ septal [cm/s]	9.0 (7.5; 11.0)	3.0	18.0
E′ lateral [cm/s]	10.5 (8.0; 13.0)	5.0	20.0
E/E′ septal	7.44 (6.0; 9.0)	3.75	32.0
E/E′ lateral	6.32 (5.42; 8.16)	2.86	26.67
E/E′ average	6.85 (5.73; 8.44)	3.54	29.33

Abbreviations: IVS—interventricular septum thickness; PWT—posterior wall thickness; LV EDD—left ventricular end-diastolic diameter; LV ESD—left ventricular end-systolic diameter; LVM—left ventricular mass; LVMI—left ventricular mass index; RWT—relative wall thickness; LV SV—left ventricular stroke volume; LV EF—left ventricular ejection fraction; LV FS—left ventricular fractional shortening; LA—left atrial dimension; LAV—left atrial volume; LAVI—left atrial volume index; RV D1—right ventricular diameter (basal); TAPSE—tricuspid annular plane systolic excursion; TRV_max_—peak tricuspid regurgitation velocity; AcT—acceleration time (pulmonary valve blood flow); E—mitral inflow E-wave velocity; A—mitral inflow A-wave velocity; S′ septal—septal S′ velocity (tissue Doppler echocardiography); S′ lateral—lateral S′ velocity (tissue Doppler echocardiography); E′ septal—early diastolic mitral annular velocity measured at the septal wall of the heart; E′ lateral—early diastolic mitral annular velocity measured at the lateral wall of the heart; E/E′ average—mean value of E/E′ calculated laterally and septally.

**Table 4:** Correlations between echocardiographic parameter values and the number of metabolic syndrome components. Significant results are presented in red.

Parameter	N	R	*p*
Left ventricle parameters			
IVS	100	0.286	0.004
PWT	100	0.235	0.019
LV EDD	100	0.337	<0.001
LV ESD	100	0.324	0.001
LVM	100	0.406	<0.001
LVMI	100	0.248	0.013
RWT	100	−0.125	0.215
LV SV	88	0.244	0.022
LV EF	100	−0.261	0.009
LV FS	100	−0.105	0.299
S′ septal	99	−0.254	0.011
S′ lateral	99	−0.27	0.007
Left atrium parameters			
LA	100	0.493	<0.001
LAV	99	0.367	<0.001
LAVI	99	0.273	0.007
Right ventricle parameters			
RV D1	100	0.389	<0.001
TAPSE	97	−0.022	0.834
Diastolic function parameters			
TRV_max_	76	−0.055	0.637
AcT	100	−0.204	0.042
E	100	−0.309	0.002
A	97	0.181	0.076
E/A	97	−0.313	0.002
E′ septal	100	−0.393	<0.001
E′ lateral	100	−0.449	<0.001
E/E′ septal	100	0.099	0.328
E/E′ lateral	100	0.195	0.052
E/E′ average	100	0.154	0.124

Abbreviations: IVS—interventricular septum thickness; PWT—posterior wall thickness; LV EDD—left ventricular end-diastolic diameter; LV ESD—left ventricular end-systolic diameter; LVM—left ventricular mass; LVMI—left ventricular mass index; RWT—relative wall thickness; LV SV—left ventricular stroke volume; LV EF—left ventricular ejection fraction; LV FS—left ventricular fractional shortening; LA—left atrial dimension; LAV—left atrial volume; LAVI—left atrial volume index; RV D1—right ventricular diameter (basal); TAPSE—tricuspid annular plane systolic excursion; TRV_max_—peak tricuspid regurgitation velocity; AcT—acceleration time (pulmonary valve blood flow); E—mitral inflow E-wave velocity; A—mitral inflow A-wave velocity; S′ septal—septal S′ velocity (tissue Doppler echocardiography); S′ lateral—lateral S′ velocity (tissue Doppler echocardiography); E′ septal—early diastolic mitral annular velocity measured at the septal wall of the heart; E′ lateral—early diastolic mitral annular velocity measured at the lateral wall of the heart; E/E′ average—mean value of E/E′ calculated laterally and septally.

**Table 5:** Data of the multivariate analysis model significance test for describing echocardiographic parameters. The model was adjusted for age, sex, and body mass index (BMI). Significant results are presented in red.

Parameter	Multiple R^2^	*p*
Left ventricle parameters		
LV EDD	0.302	<0.001
LV ESD	0.256	<0.001
LVM	0.381	<0.001
LVMI	0.202	<0.001
RWT	0.084	0.077
LV FS	0.022	0.711
Left atrium parameters		
LA	0.374	<0.001
LAV	0.312	<0.001
LAVI	0.212	<0.001
Right ventricle parameters		
log(RV D1)	0.255	<0.001
Diastolic function parameters		
TRV_max_	0.165	0.011
AcT	0.051	0.281
log(E)	0.104	0.032
log(A)	0.278	<0.001
log(E′ septal)	0.429	<0.001
log(E′ lateral)	0.443	<0.001
log(E/E′ septal)	0.202	<0.001
log(E/E′ lateral)	0.177	<0.001
log(E/E′ average)	0.204	<0.001

Abbreviations: LV EDD—left ventricular end-diastolic diameter; LV ESD—left ventricular end-systolic diameter; LVM—left ventricular mass; LVMI—left ventricular mass index; RWT—relative wall thickness; LV FS—left ventricular fractional shortening; LA—left atrial dimension; LAV—left atrial volume; LAVI—left atrial volume index; RV D1—right ventricular diameter (basal); TRV_max_—peak tricuspid regurgitation velocity; AcT—acceleration time (pulmonary valve blood flow); E—mitral inflow E-wave velocity; A—mitral inflow A-wave velocity; E′ septal—early diastolic mitral annular velocity measured at the septal wall of the heart; E′ lateral—early diastolic mitral annular velocity measured at the lateral wall of the heart; E/E′ average—mean value of E/E′ calculated laterally and septally.

**Table 6:** Data of the multivariate analysis model significance test for describing echocardiographic parameters (adjusted for age, sex, and body mass index).

Parameter	*β*	95% CI	*p*
Left ventricle parameters			
LV EDD	0.088	−0.154; 0.33	0.472
LV ESD	0.095	−0.154; 0.346	0.45
LVM	0.14	−0.089; 0.368	0.228
LVMI	0.106	−0.153; 0.365	0.418
RWT	0.089	−0.189; 0.366	0.526
LV FS	−0.026	−0.313; 0.26	0.856
Left atrium parameters			
LA	0.137	−0.092; 0.367	0.237
LAV	0.113	−0.129; 0.355	0.356
LAVI	0.077	−0.182; 0.336	0.558
Right ventricle parameters			
log(RV D1)	0.095	−0.155; 0.345	0.453
Diastolic function parameters			
TRV_max_	−0.103	−0.394; 0.188	0.483
AcT	−0.196	−0.478; 0.087	0.172
log(E)	−0.089	−0.363; 0.186	0.523
log(A)	−0.035	−0.286; 0.215	0.779
log(E′ septal)	−0.178	−0.397; 0.041	0.111
log(E′ lateral)	−0.172	−0.389; 0.044	0.117
log(E/E′ septal)	0.11	−0.149; 0.369	0.401
log(E/E′ lateral)	0.089	−0.174; 0.352	0.505
log(E/E′ average)	0.105	−0.153; 0.364	0.421

Abbreviations: LV EDD—left ventricular end-diastolic diameter; LV ESD—left ventricular end-systolic diameter; LVM—left ventricular mass; LVMI—left ventricular mass index; RWT—relative wall thickness; LV FS—left ventricular fractional shortening; LA—left atrial dimension; LAV—left atrial volume; LAVI—left atrial volume index; RV D1—right ventricular diameter (basal); TRV_max_—peak tricuspid regurgitation velocity; AcT—acceleration time (pulmonary valve blood flow); E—mitral inflow E-wave velocity; A—mitral inflow A-wave velocity; E′ septal—early diastolic mitral annular velocity measured at the septal wall of the heart; E′ lateral—early diastolic mitral annular velocity measured at the lateral wall of the heart; E/E′ average—mean value of E/E′ calculated laterally and septally.

**Table 7:** Data of the multivariate analysis model significance test for describing selected echocardiographic parameters in patients with and without metabolic syndrome analyzed separately. The model was standardized for age, sex, and body mass index (BMI). Significant results are presented in red.

Parameter	Patients Without Metabolic Syndrome		Patients with Metabolic Syndrome	
	Multiple R^2^	*p*	Multiple R^2^	*p*
LAV	0.353	<0.001	0.256	0.016
LAVI	0.306	0.001	0.16	0.127

Abbreviations: LAV—left atrial volume; LAVI—left atrial volume index.

**Table 8:** Data from the multivariate analysis for describing echocardiographic parameters (adjusted for age, sex, and body mass index) in patients with and without metabolic syndrome analyzed separately. Significant results are presented in red.

Parameter	Patients Without Metabolic Syndrome			Patients with Metabolic Syndrome		
	*β*	95% CI	*p*	*β*	95% CI	*p*
LAV	0.44	0.111; 0.768	0.01	−0.06	−0.36; 0.241	0.69
LAVI	0.361	0.021; 0.702	0.038	NA	NA	NA

Abbreviations: LAV—left atrial volume; LAVI—left atrial volume index; NA—not applicable.

## Data Availability

The data presented in this study are available on request from the corresponding author due to privacy concerns.
